# Egg Consumption, Cardiovascular Disease and Cardiometabolic Risk Factors: The Interaction with Saturated Fatty Acids. Results from the ATTICA Cohort Study (2002–2012)

**DOI:** 10.3390/nu14245291

**Published:** 2022-12-12

**Authors:** Matina Kouvari, Evangelia Damigou, Matilda Florentin, Rena I. Kosti, Christina Chrysohoou, Christos S. Pitsavos, Demosthenes B. Panagiotakos

**Affiliations:** 1 Department of Nutrition and Dietetics, School of Health Science and Education, Harokopio University, 17272 Athens, Greece; 2 Discipline of Nutrition and Dietetics, Faculty of Health, University of Canberra, Canberra, ACT 2601, Australia; 3 Functional Foods and Nutrition Research (FFNR) Laboratory, University of Canberra, Bruce, ACT 2617, Australia; 4 Department of Internal Medicine, School of Medicine, University of Ioannina, 45221 Ioannina, Greece; 5 Department of Nutrition and Dietetics, School of Physical Education, Sport Science and Dietetics, University of Thessaly, 38221 Trikala, Greece; 6 First Cardiology Clinic, School of Medicine, University of Athens, 11527 Athens, Greece

**Keywords:** nutrition, dietary cholesterol, saturated fatty acids, heart disease, metabolic syndrome

## Abstract

Purpose: To examine the association of egg intake with 10-year risk of cardiovascular disease (CVD) and other cardiometabolic risk factors in a sample of individuals of Mediterranean origin. Methods: In 2001–2002, *n* = 1514 men and *n* = 1528 women (>18 years old) from the greater Athens area, Greece, were enrolled. Information on any egg intake, eaten as a whole, partly or in recipes was assessed via a validated semi-quantitative food frequency questionnaire. Follow-up for CVD evaluation (2011–2012) was achieved in *n* = 2020 participants (*n* = 317 CVD cases). Results: Ranking from lowest (<1 serving/week) to intermediate (1–4 servings/week) and high (4–7 servings/week) egg consumption tertiles, lower CVD incidence was observed (18%, 9% and 8%, respectively, *p*-for-trend = 0.004). Unadjusted analysis revealed that 1–3 eggs/week and 4–7 eggs/week were associated with a 60% and 75%, respectively, lower risk of developing CVD compared with the reference group (<1 egg/week). When adjusting for sociodemographic, lifestyle and clinical factors, significance was retained only for 1–3 eggs/week (hazard ratio (HR) = 0.53, 95% confidence interval (95% CI) = 0.28, 1.00). When total saturated fatty acid (SFA) intake was taken into account, this inverse association was non-significant. Multi-adjusted analysis revealed that in participants of low SFA intake, 1 serving/day increase in egg intake resulted in 45% lower risk of developing CVD. In the case of higher SFA consumption, only 1–3 eggs/week seemed to protect against CVD (HR = 0.25, 95% CI = 0.07, 0.86). In the case of intermediate cardiometabolic disorders, no significant trend was observed. Conclusions: Overall dietary habits principally in terms of SFA intake may be detrimental to define the role of eggs in cardiac health.

## 1. Introduction

The association of egg consumption—principally due to its high cholesterol content—with cardiovascular disease (CVD) remains controversial despite decades of research [1]. Historically, nutrition guidelines for reducing CVD risk and achieving optimal plasma lipoprotein profiles have included recommendations to limit dietary cholesterol [2]. However, contemporary guidelines and position statements for CVD risk reduction from the “2020–2025 Dietary Guidelines for Americans” [3], the American Heart Association (AHA) [4] and the European Society of Cardiology [5] have not issued explicit guidance for cholesterol, even more so when it comes to the most important representative of this dietary element, i.e., eggs. An in-depth analysis of the topic has been recently launched by the AHA in the context of a scientific statement on dietary cholesterol and cardiometabolic risk [6]. The statement concluded that “examining the association between dietary cholesterol and foods with high content in dietary cholesterol remains challenging due to the given limitations when isolating one nutrient or one food group” [6].

The contradicting outcomes even on the basis of meta-analyses—presenting from positive to negative associations—warrant obtaining stronger evidence for a possible protective role of moderate weekly egg consumption compared with no intake in terms of CVD, as well as examining this association in diverse environments (e.g., US, Mediterranean, etc.) with different dietary habits [1,7,8]. Thereby, the present work examined the association of egg intake with the risk of developing CVD and other intermediate cardiometabolic conditions (i.e., type II diabetes, hypercholesterolemia, hypertension) within a decade in a sample of individuals of Mediterranean origin. Two a priori research hypotheses were examined: (i) moderate egg consumption is inversely associated with 10-year cardiometabolic risk compared with no consumption due to the beneficial nutrients of eggs, as well as the general dietary pattern adapted in the study population (i.e., Mediterranean diet) and (ii) this association is influenced by the participants’ overall dietary habits.

## 2. Materials and Methods

### 2.1. Study Sample

The ATTICA study is a prospective, observational cohort investigation which was initiated in 2001 [9]. At baseline (2001–2002), *n* = 3042 apparently healthy volunteers residing in the greater metropolitan Athens area, Greece, agreed to participate (75% participation rate). Of the enrolled participants, *n* = 1514 (49.8%) were men (46 ± 13 years) and *n* = 1528 (50.2%) were women (45 ± 14 years). During baseline examination, a detailed clinical evaluation was performed by trained physicians; all participants were free of CVD and other chronic diseases, according to the study protocol. For the scope of the present work, we used the *n* = 2020 participants with complete CVD evaluation in the follow-up assessment.

### 2.2. Bioethics

The study was carried out in accordance with the Declaration of Helsinki (1989) of the World Medical Association. The ATTICA study was approved by the Institutional Ethics committee of Athens Medical School (#017/1.5.2001) and all participants were informed about the aims and procedures and agreed to participate providing written consent.

### 2.3. Dietary Assessment

Dietary assessment was based on a validated semi-quantitative FFQ kindly provided by the Laboratory of Epidemiology, School of Medicine, University of Athens [10]. The questionnaire assessed usual dietary intake of 156 foods and beverages commonly consumed in Greece, with 7 non-overlapping response categories. Photographs assisted the responders to define the portion sizes of several foods which were included in the questionnaire. In particular, consumption of non-refined cereals and products (such as whole grain bread, pasta, rice, etc.), vegetables, legumes, fruit, dairy products (such as cheese, yoghurt, milk), nuts, potatoes, eggs, sweets, fish, poultry, red meat and meat products, use of olive oil in cooking, as well as coffee and alcohol drinking were measured as an average per week during the past year. The frequency of consumption was quantified in terms of the number of times a month a food was consumed in small, medium or large portion sizes. Information on any egg intake, eaten as a whole, partly or in recipes was assessed via the FFQ. The total egg intake in grams per individual was calculated and then transformed to egg equivalents, i.e., (1 egg = 50 g). Participants were separated into three levels of consumption categories, i.e., <1 egg/week, 1–3 eggs/week and 4–7 eggs/week. Total energy and saturated fatty acid intake were calculated using international food databases and the Greek food composition tables. Alcohol consumption was recorded as daily ethanol intake of 100 mL wineglasses adjusted for 12% ethanol concentration.

### 2.4. Other Measurements

The biochemical evaluation was carried out in one laboratory, following the criteria of the World Health Organization Reference Laboratories. C-reactive protein (CRP) and serum amyloid A were assayed by particle-enhanced immunonephelometry. Interleukin-6 (IL-6) was measured with high-sensitivity enzyme-linked immunoassay. The intra- and interassay coefficients of variation were <5% for CRP and serum amyloid A and <10% for IL-6. Plasma fibrinogen levels were measured using automatic nephelometry. Homocysteine was measured using the pulsar fluorescence method. The intra- and interassay coefficients of variation for fibrinogen and homocysteine did not exceed 9%. The ELISA method was used for the quantitative determination of human tumor necrosis factor (TNF-a) (in duplicate) in serum samples of the participants by the Quantikine HS/human TNF-alpha immunoassay kit (R & D Systems Inc., Minneapolis, MN, USA). Insulin resistance was assessed by calculation of a Homeostatic Model Assessment of Insulin Resistance (HOMA-IR) approach (glucose (mmol/L) × insulin (μU/mL)/22.5).

### 2.5. Endpoint and Follow-Up Evaluation

#### 2.5.1. Hard Endpoints

The combined endpoint studied in this work was the development of a fatal or non-fatal CVD event. A CVD event was defined as the development of: acute myocardial infarction, unstable angina or other identified forms of ischemia (WHO-ICD coding 410–414.9, 427.2, 427.6), heart failure of different types and chronic arrhythmias (WHO-ICD coding 400.0–404.9, 427.0–427.5, 427.9–) or stroke (WHO-ICD coding 430–438).

#### 2.5.2. Intermediate Cardiometabolic Conditions

Diagnosis of diabetes was based on American Diabetes Association criteria; participants who had fasting blood glucose >126 mg/dL during the examination or who reported the use of antidiabetic medications were defined as having diabetes. Diagnosis of hypertension and hypercholesterolemia was based on ESC criteria; hypertension was defined as blood pressure ≥140/90 mmHg or use of antihypertensive medications and hypercholesterolemia as total cholesterol ≥200 mg/dL or use of lipid-lowering agents. Participants who either did not provide biological samples, clinical evaluation or both—those who were reached only by telephone—were asked whether they had been diagnosed by a physician. Patients diagnosed with the aforementioned conditions at baseline and those with no data at the 10-year follow-up were excluded from the analysis.

For participants who died during follow-up, information was retrieved from relatives and death certificates.

#### 2.5.3. Statistical Analysis

Categorical variables are presented as absolute (*n*) and relative frequencies (%). Continuous variables are presented as mean values ± standard deviation. Associations between normally distributed variables and the egg consumption intake categories were evaluated through one-way analysis of variance. Whether these variables were normally distributed was tested through P-P plot and equality of variances through Levene’s test. For non-normally distributed variables, Kruskal–Wallis test was used. Associations between categorical variables and the egg consumption intake categories were tested with the chi-squared test. Hazard ratios and their corresponding 95% Confidence Intervals (95% CIs) for egg consumption intake in relation to the examined endpoints within the decade were evaluated through multivariable Cox-regression analysis in the total sample. A subgroup analysis was performed in order to evaluate the relationship between egg consumption intake and the examined 10-year endpoints according to the level of saturated fatty acid intake (calories attributed to saturated fatty acid intake <10% vs. ≥10% of total daily energy intake). STATA software, version 14 (MP & Associates, Sparta, Greece), was used for all statistical analyses. Two-sided level of significance was set at *p* < 0.05.

## 3. Results

Baseline sociodemographic, anthropometric, lifestyle, clinical factors as well as biochemical markers (inflammation/coagulation, glucose/insulin homeostasis, renal function) of the ATTICA study subjects across the egg consumption categories are presented in Table 1. Ranking from the lowest (i.e., <1 egg/week) to the highest level of consumption (4–7 eggs/weeks), significantly lower adherence to the Mediterranean diet (via the MedDietScore) (*p =* 0.002) and higher intake of saturated fatty acids (all *p* for trend < 0.001) were observed. The subgroup of participants reporting <1 egg/week intake presented the worst metabolic profile in terms of waist circumference, history of hypertension and hypercholesterolemia (all *p* for trend < 0.05). However, no significant differences were observed when the 2nd egg consumption category (i.e., 1–3 eggs/week) was compared with the 3rd one (4–7 eggs/week). Similarly, focusing on biochemical markers, ranking from lowest to highest egg consumption category, interleukin-6 (*p* for trend = 0.009) and fibrinogen (*p* for trend = 0.004) were reduced.

Results from unadjusted analysis regarding the association between the onset of hard and intermediate CVD-related endpoints within the 10-year follow-up period are presented in Table 2. Concerning fatal/non-fatal CVD events, participants assigned in the lowest egg consumption category presented around twice as high CVD event rate compared with their counterparts in higher level-of-consumption categories (*p =* 0.004). Concerning the other intermediate cardiometabolic conditions (hypertension, diabetes mellitus, hypercholesterolemia), no significant trends were found (all *p >* 0.05).

Multi-adjusted Cox regression models to evaluate the association between egg consumption level (as a continuous as well as a categorical variable), and 10-year incidence of CVD and other cardiometabolic factors (hypertension, diabetes mellitus, hypercholesterolemia) are presented in Table 3. Unadjusted analysis revealed that egg consumption at a level of 1–3 eggs/week and 4–7 eggs/week was associated with 60% and 75% lower risk of developing CVD within the decade compared with their counterparts that reported <1 egg/week consumption. When adjusting for sociodemographic, lifestyle and clinical factors (model 3), the aforementioned associations were retained only in the case of moderate level of consumption (HR = 0.53, 95% CI = 0.28, 1.00). In model 4, where total saturated fatty acid intake was taken into account, this inverse association remained, yet was non-significant. For all the cardiometabolic conditions (hypertension, diabetes mellitus, hypercholesterolemia), no significant association was found (all *p* < 0.05).

Interaction analysis revealed a significant interaction between egg consumption and saturated fatty acid intake on the 10-year CVD onset (*p* for interaction = 0.01). Stratified analysis by saturated fatty acid intake is presented in Table 4. Multi-adjusted analysis revealed that in those participants with low saturated fatty acid intake, increasing the level of consumption by 1 egg on a daily basis resulted in a 45% lower risk of developing CVD within the decade. Additionally, only the highest level of egg consumption, i.e., 4–7 eggs/week, seemed to protect against CVD, resulting in a 75% lower risk compared with the reference group (i.e., <1 egg/week). On the other side, in the case of participants with higher level of consumption in saturated fatty acids, only the level of consumption of 1–3 eggs/week seemed to protect against CVD, with participants assigned in this group having a 71% lower risk compared with their lowest level of consumption counterparts. When the same stratified analyses were repeated in relation to intermediate cardiometabolic conditions, no significant associations were observed.

## 4. Discussion

The outcomes of the present work suggest that egg consumption even on a daily basis may belong to the food habits that protect against major CVD events in the long-term, yet only in the context of a generally plant-based dietary pattern of low saturated fatty acid content. Of interest, no significant associations were observed with intermediate cardiometabolic conditions including type II diabetes. Despite the limitations of this study due to its observational nature, the findings here add to the existing controversies in this field even after many years of research.

Eggs remain one of the most “controversial” foods due to their saturated fatty acid (3 g/100 g) and cholesterol content (370 mg/100 g) along with their composition, which is rich in high quality protein, iron, fat-soluble vitamins, minerals and carotenoids [11]. Current literature demonstrates rather heterogeneous results, principally due to previous work that suggested a positive association between egg intake and diabetes onset and progression [12]. A recent meta-analysis of individual participant data from ethnically diverse US cohorts concluded that half an egg per day was associated with a 6% increase in CVD risk [8]. A pooled analysis of three US cohort studies, in a sample with a consumption range of 1–5 eggs/week, showed no significant association with CVD risk. This is in line with several meta-analyses of cohorts where a moderate egg consumption (up to 1 egg/day) did not seem to alter CVD risk [13,14,15]. Another meta-analysis revealed that even >1 egg/day compared with ≤1 egg/day was not associated with overall CVD, while it resulted in a significant reduction in coronary heart disease (CHD) risk [16]. Of interest, a meta-analysis of 21 studies published in 2021 showed decreased overall CVD risk in the case of consumption of up to 1 egg/day; yet once studies for CHD were isolated, only up to 2 eggs/week was associated with lower CHD risk compared with no/rare consumption [17]. A couple of months ago (April and May 2022), two pooled analyses of 32 [18] and 55 [19] studies, respectively, of low to moderate quality were launched examining the role of egg intake on all-cause and cause-specific mortality. Even if no statistically or clinically significant association was observed in relation to CVD and other cardiometabolic disorders, a positive association with cancer mortality was revealed [18,19]. Hence, these studies suggested a consumption of <1 egg/day in the context of a generally healthy diet due to the competing risk of cancer [18,19].

In the context of rather contradicting literature, our study with a sample of individuals of Mediterranean origin suggests that eggs may even have a protective role in CVD onset, yet only in the case of adherence to a generally healthy dietary pattern characterized by low consumption of saturated fatty acids overall. This is in line with the findings from a cohort study implemented in Italy, where the aggravating effect of increased egg consumption was principally mediated by the overall intake of dietary cholesterol [20]. Many previous works have commented on the mediating role of overall dietary patterns when examining their effect on cardiometabolic health principally due to the diversity in egg consumption patterns (i.e., cooking methods, inclusion in recipes, etc.). To discover these kinds of inconsistencies, the latest meta-analyses in this field present the results separately for European and US cohorts. For instance, a very recent meta-analysis of prospective studies suggested 18% higher diabetes risk in US populations [21]. Yet, no significance was reached in European studies [13]. Similarly, in the subgroup analysis of geographic regions from the Alpha-Tocopherol, Beta-Carotene Cancer Prevention (ATBC) cohort study accompanied by a meta-analysis of other cohort studies revealed that 50 g egg consumed daily was associated with a higher risk of CVD in US cohorts [7]. On the other side, in the case of European cohorts, a borderline significance was observed with the same amount of egg consumption. This probably implies the confounding effect of other dietary factors, including culinary techniques [7].

### 4.1. Limitations

The present findings should be interpreted with caution given the observational study design employed. Despite using country specific validated FFQ [10], some measurement error is inevitable, which in a prospective study would most often attenuate findings toward null. Only baseline FFQ responses were included in the analysis, which may challenge the consistency of dietary habits within the decade; however, based on the information retrieved from a section of individuals at follow-up examination, no differences were observed in consumption of all major food groups and beverages as well as level of adherence to Mediterranean diet [22].

### 4.2. Conclusions

A consumption level of 2–4 eggs/week is the current recommendation of most health bodies and international guidelines. The findings presented here seem to be overall in line with this recommendation. In addition to this, our study—in line with the most recent literature—suggest an increase in this limit in an adult population who follow a plant-based dietary pattern with low saturated fatty acid content. Nevertheless, considering the latest findings on the linear association between egg intake and major non-CVD outcomes, such as cancer mortality, caution should be taken especially for high-risk individuals. What is more, other sources of dietary cholesterol, such as meat, shellfish, full-fat dairy products and so on, should be examined in comparison with egg intake to adjust the dietary recommendation of primary CVD prevention accordingly.

## Figures and Tables

**Table 1:** Baseline sociodemographic, clinical, anthropometric, biochemical and lifestyle characteristics of subjects from the ATTICA study according to level of egg consumption (*n* = 2020).

Baseline Characteristics	Egg Consumption Categories			
	Never/Rare Consumption (<1 Egg/Week)	Low/Moderate Consumption (1–3 Eggs/Week)	Frequent Consumption (4–7 Eggs/Week)	*p*-Value
*n*	443	839	738	*-*
Sociodemographic factors				
Age, years	42 (11)	40 (10)	38 (10)	0.523
Male sex, %	57	54	52	0.519
Anthropometric factors				
Body mass index, kg/m^2^	26.17 (4.39)	26.10 (4.50)	25.41 (4.60)	0.736
Waist circumference, cm	92 (21)	89 (22)	88 (23)	0.052
Lifestyle factors				
Physical activity, %	34.08	36.17	39.25	0.419
MedDietScore, range 0–55	23.9 (3.0)	26.6 (3.2)	27.0 (3.3)	0.002
Total daily saturated fatty acid intake, g/day	27.40 (18.20)	30.31 (19.20)	38.52 (23.33)	<0.001
Current smoking, %	44	46	45	0.918
Clinical factors				
History of hypertension, %	33	27	23	0.029
History of diabetes mellitus, %	7	4	3	0.155
History of hypercholesterolemia, %	44	31	23	<0.0001
Family CVD history, %	38	34	36	0.706
Inflammation/coagulation markers				
C-Reactive Protein, mg/L	1.12 (1.91)	1.02 (1.80)	0.84 (1.85)	0.092
Interleukin 6, pg/dL	1.39 (0.30)	1.34 (0.32)	1.29 (0.30)	0.009
Tumor necrosis factor-alpha, pg/mL	6.04 (3.32)	6.39 (3.07)	5.91 (3.25)	0.143
Amyloid A, mg/L	2.8 (3)	2.9 (3)	2.6 (2.7)	0.639
Homocysteine, μmol/L	10.22 (3.97)	10.38 (4.28)	10.41 (4.15)	0.527
Fibrinogen, mg/dL	309 (79)	299.5 (81.5)	289 (69)	0.004
Glucose/insulin homeostasis markers				
HOMA-IR	2.91 (1.14)	2.81 (0.96)	2.76 (0.93)	0.08
Renal function markers				
Creatinine clearance, mL/min/1.73 m^2^	94.02 (32.00)	95.95 (33.82)	95.03 (32.39)	0.648

Data are presented as mean ± standard deviation (SD) or median (interquartile range) if normality was not met. *p*-values were obtained using one-way ANOVA for the normally distributed variables (age, body mass index), Kruskal–Wallis test for the remaining quantitative variables and chi-squared test for categorical variables. Abbreviations: Cardiovascular disease (CVD); homeostatic model assessment of insulin resistance (HOMA-IR).

**Table 2:** Ten-year incidence of first fatal/non-fatal cardiovascular disease incidence and intermediate cardiometabolic conditions according to egg level of consumption, in the ATTICA study participants (*n* = 2020).

10-Year Endpoint, %	Egg Consumption Categories			*p*-Value
	Never/Rare Consumption (<1 Egg/Week)	Low/Moderate Consumption (1–3 Eggs/Week)	Frequent Consumption (4–7 Eggs/Week)	
Fatal/non-fatal CVD event	18	9	8	0.004
Hypertension	24	20	15	0.174
Hypercholesterolemia	36	31	31	0.724
Diabetes mellitus	7	9	5	0.221

*p*-values were obtained using chi-squared test. Abbreviations: Cardiovascular disease (CVD).

**Table 3:** Sensitivity analyses to evaluate the association of egg consumption intake (portions/week) with 10-year cardiometabolic endpoints of cardiovascular disease, hypertension, hypercholesterolemia, diabetes mellitus (*n* = 2020).

	CVD	Hypertension	Hypercholesterolemia	Diabetes Mellitus	
*n*, cases	2020/317	1154/320	1044/352	1485/191	
	HR (95% CI)	HR (95% CI)	HR (95% CI)	HR (95% CI)	Models adjusted for
Model with egg consumption intake as continuous variable					Crude model
per 1 egg/day	0.80 (0.62, 1.05)	0.81 (0.61, 1.07)	1.00 (0.81, 1.23)	0.70 (0.46, 1.05)	
Model with egg consumption intake as categorical variable					
Never/rare consumption (<1 egg/week)	Ref	Ref	Ref	Ref	
Low/moderate consumption (1–3 eggs/week)	**0.40 (0.21, 0.75)**	0.78 (0.41, 1.49)	0.8 (0.44, 1.43)	1.25 (0.53, 2.93)	
Frequent consumption (4–7 eggs/week)	**0.25 (0.08, 0.75)**	0.53 (0.27, 1.06)	0.80 (0.45, 1.44)	0.63 (0.24, 1.66)	
Model with egg consumption intake as continuous variable					Model 1: Age, sex
per 1 egg/day	0.94 (0.73, 1.23)	0.87 (0.65, 1.16)	1.04 (0.84, 1.29)	0.86 (0.59, 1.26)	
Model with egg consumption intake as categorical variable					
Never/rare consumption (<1 egg/week)	Ref	Ref	Ref	Ref	
Low/moderate consumption (1–3 eggs/week)	**0.53 (0.29, 0.95)**	1.04 (0.51, 2.09)	0.91 (0.49, 1.68)	1.92 (0.78, 4.76)	
Frequent consumption (4–7 eggs/week)	0.56 (0.29, 1.06)	0.71 (0.34, 1.50)	0.96 (0.52, 1.76)	1.05 (0.38, 2.90)	
Model with egg consumption intake as continuous variable					Model 2: Model 1 plus body mass index, physical activity, current smoking, MedDietScore
per 1 egg/day	0.97 (0.74, 1.26)	0.84 (0.62, 1.13)	1.03 (0.83, 1.28)	0.87 (0.58, 1.31)	
Model with egg consumption intake as categorical variable					
Never/rare consumption (<1 egg/week)	Ref	Ref	Ref	Ref	
Low/moderate consumption (1–3 eggs/week)	**0.50 (0.27, 0.93)**	1.14 (0.54, 2.41)	0.94 (0.50, 1.76)	1.74 (0.68, 4.45)	
Frequent consumption (4–7 eggs/week)	0.57 (0.30, 1.10)	0.75 (0.34, 1.65)	0.94 (0.50, 1.76)	1.09 (0.38, 3.12)	
Model with egg consumption intake as continuous variable					Model 3: Model 2 plus history of (hypertension, hypercholesterolemia and diabetes mellitus), family history of CVD
per 1 egg/day	0.94 (0.71, 1.25)	0.81 (0.59, 1.11)	1.03 (0.82, 1.30)	0.87 (0.57, 1.33)	
Model with egg consumption intake as categorical variable					
Never/rare consumption (<1 egg/week)	Ref	Ref	Ref	Ref	
Low/moderate consumption (1–3 eggs/week)	**0.53 (0.28, 1.00)**	1.19 (0.56, 2.52)	0.97 (0.50, 1.87)	1.83 (0.70, 4.77)	
Frequent consumption (4–7 eggs/week)	0.57 (0.29, 1.15)	0.74 (0.33, 1.67)	0.90 (0.46, 1.73)	1.13 (0.38, 3.41)	
Model with egg consumption intake as continuous variable					Model 4: Model 3 plus total daily saturated fatty acid intake
per 1 egg/day	1.01 (0.76, 1.36)	0.76 (0.54, 1.07)	1.22 (0.85, 1.76)	0.90 (0.58, 1.38)	
Model with egg consumption intake as categorical variable					
Never/rare consumption (<1 egg/week)	Ref	Ref	Ref	Ref	
Low/moderate consumption (1–3 eggs/week)	0.54 (0.28, 1.03)	1.16 (0.54, 2.47)	0.65 (0.26, 1.62)	1.86 (0.71, 4.85)	
Frequent consumption (4–7 eggs/week)	0.64 (0.31, 1.32)	0.67 (0.29, 1.54)	1.67 (0.62, 4.5)	1.22 (0.40, 3.73)	

HRs and their corresponding 95% CIs were obtained from Cox regression analysis. Bold indicates statistically significant outcomes, i.e., *p* < 0.05. Abbreviations: Cardiovascular disease (CVD); confidence interval (CI); hazard ratio (HR).

**Table 4:** Stratified analyses by saturated fatty acid intake.

	CVD		Hypertension		Hypercholesterolemia		Diabetes Mellitus	
*n*, Cases	2020/317		1154/320		1044/352		1485/191	
	Low saturated fat intake *	High saturated fat intake *	Low saturated fat intake *	High saturated fat intake *	Low saturated fat intake *	High saturated fat intake *	Low saturated fat intake *	High saturated fat intake *
	HR (95% CI)	HR (95% CI)	HR (95% CI)	HR (95% CI)	HR (95% CI)	HR (95% CI)	HR (95% CI)	HR (95% CI)
Model with egg consumption intake as continuous variable								
per 1 egg/day	**0.55** **(0.32, 0.93)**	1.09 (0.61, 1.95)	0.68 (0.38, 1.19)	0.84 (0.46, 1.59)	0.97 (0.62, 1.52)	0.97 (0.59, 1.58)	1.11 (0.54, 2.30)	0.89 (0.39, 2.00)
Model with egg consumption intake as categorical variable								
Never/rare consumption (<1 egg/week)	Ref	Ref	Ref	Ref	Ref	Ref	Ref	Ref
Low/moderate consumption (1–3 eggs/week)	0.68 (0.30, 1.49)	**0.29** **(0.08, 1.00)**	1.41 (0.55, 3.61)	0.76 (0.19, 2.96)	0.81 (0.34, 1.95)	1.08 (0.37, 3.15)	1.61 (0.46, 5.64)	1.65 (0.30, 9.09)
Frequent consumption (≥4 eggs/week)	**0.25** **(0.07, 0.86)**	0.87 (0.29, 2.57)	0.37 (0.09, 1.37)	0.71 (0.19, 2.63)	0.92 (0.37, 2.25)	0.98 (0.34, 2.80)	1.21 (0.26, 5.62)	0.94 (0.16, 5.53)

* The cut-off value of 10% of total daily energy intake attributed to saturated fatty acids was defined, i.e., low level of intake: <10% vs. high level of intake: ≥10%HRs and their corresponding 95% CIs were obtained from Cox regression analysis. All models were adjusted for age, sex, body mass index, physical activity, current smoking, MedDietScore, history of (hypertension, hypercholesterolemia and diabetes mellitus) and family history of CVD. Bold indicates statistically significant outcomes, i.e., *p* < 0.05. Abbreviations: Cardiovascular disease (CVD); confidence interval (CI); hazard ratio (HR).

## Data Availability

Data are not available for confidentiality reasons.
